# Comparative Analysis of Targeted RNA-Seq and Optical Genome Mapping for Detecting Gene Rearrangements in Acute Leukemia

**DOI:** 10.3390/cancers17213458

**Published:** 2025-10-28

**Authors:** Chi Young Ok, Guilin Tang, Sanam Loghavi, Shimin Hu, Qing Wei, Andres E. Quesada, Mark J. Routbort, Rashmi Kanagal-Shamanna, C. Cameron Yin, Iman Sarami, Sofia Garces, Nitin K. Agarwal, Raja Luthra, Hong Fang, Fatima Zahra Jelloul, Julian Bryan, L. Jeffrey Medeiros, Keyur P. Patel, Gokce A. Toruner

**Affiliations:** Department of Hematopathology, The University of Texas MD Anderson Cancer Center, Houston, TX 77030, USA; cok@mdanderson.org (C.Y.O.);

**Keywords:** optical genome mapping, targeted RNA sequencing, acute leukemia

## Abstract

**Simple Summary:**

We compared the results using a 108-gene RNA-seq panel with those derived from optical genome mapping (OGM) in 467 acute leukemias. The overall concordance rate was 88.1%. For clinically relevant events (n = 234), OGM uniquely identified 15.8%, whereas RNA-seq uniquely identified 9.4%. Concordance was poor for enhancer-hijacking lesions (20.6%), including *MECOM*, *BCL11B*, and *IGH* rearrangements, many of which were not detected by RNA-seq. Conversely, RNA-seq slightly outperformed OGM for fusions arising from intrachromosomal deletions that were sometimes labeled by OGM as simple deletions. Overall, RNA-seq is more sensitive for detecting expressed chimeric fusions, whereas OGM is better for detecting cryptic, enhancer-driven events. Using both platforms provides the most comprehensive structural variant detection in acute leukemia cases.

**Abstract:**

**Background/Objectives:** Gene rearrangements involving oncogenes are major drivers in acute leukemia, influencing disease classification, prognosis, and therapeutic decision-making. Targeted RNA sequencing (RNA-Seq) panels capable of detecting intergenic and intragenic fusions across multiple genes are increasingly used in diagnostic settings. However, comparative evaluation with orthogonal technologies remains limited. **Material and Methods**: We compared the performance of a 108-gene anchored multiplex PCR (AMP)-based RNA-Seq panel with that of Optical Genome Mapping (OGM) in 467 acute leukemia cases. The cohort included 360 cases of acute myeloid leukemia (AML), 89 B-lymphoblastic leukemia (B-ALL), 12 T-lymphoblastic leukemia (T-ALL), and 6 cases of mixed phenotype acute leukemia (MPAL). **Results:** Results of both methods were concordant in 175 (74.7%) of 234 detected gene/rearrangement fusions. The concordance rate varied significantly across different leukemia types, ranging from 80.2% in B-ALL to 41.7% in T-ALL (*p* < 0.001) OGM uniquely detected 37 of 234 (15.8%) clinically relevant rearrangements, whereas RNA-Seq exclusively identified 22 of 234 (9.4%). Enhancer-hijacking lesions, including *MECOM* and *BCL11B* rearrangements, *CDK6::MNX1*, and *IGH* rearrangements, had a markedly lower concordance (20.6%) compared with all other aberrations (93.1%) (*p* < 0.001). Conversely, some gene fusions arising from intrachromosomal deletions were interpreted by OGM as simple deletions rather than rearrangements or fusions. **Conclusions:** Targeted RNA-Seq was effective for detecting chimeric fusion transcripts and showed slightly better performance in identifying fusions resulting from deletions. However, OGM was effective for detecting enhancer-hijacking events that do not generate fusion transcripts. Both methods are complementary for the workup of acute leukemia cases.

## 1. Introduction

Somatic structural variations (SVs) resulting from genomic rearrangements are critical events in the pathogenesis of acute leukemia. These alterations not only serve as key drivers of leukemogenesis but are also integral to disease classification, risk stratification, and therapeutic decision-making. As such, accurate detection of SVs is essential because they directly impact patient management.

Historically, SV detection has relied on conventional G-banded karyotyping, fluorescence in situ hybridization (FISH) at the DNA level, and reverse transcription PCR (RT-PCR) at the RNA level. These methods are effective in certain contexts, but these approaches are inherently limited in scope and resolution. Over the past two decades, advances in genomic technologies have enabled high-throughput, multiplexed analysis of SVs across the genome. At the DNA level, whole-genome sequencing [1,2,3,4,5,6,7,8,9,10,11,12,13,14,15] and optical genome mapping (OGM) [9,16,17,18,19,20,21,22,23,24,25,26,27] have emerged as powerful tools. At the RNA level, both whole-transcriptome sequencing [1,4,12,28,29,30,31,32,33] and targeted RNA sequencing (RNA-seq) [28,34,35,36,37] panels are being increasingly adopted in clinical and research settings.

Among these methods, OGM and targeted RNA-seq panels are gaining traction for the evaluation of hematologic malignancies in clinical laboratories. These platforms are commercially available at a relatively reasonable cost and offer user-friendly, standardized bioinformatics pipelines that facilitate implementation. Despite their growing adoption, comparative real-world data on the comparative performance of these two methods for SV detection in diagnostic settings are scant.

There is a need for performing systematic head-to-head comparative studies between different laboratory methodologies targeting the same entities. Multi-modal testing is a fact of life in genetic testing. When there is a discrepancy between different methodologies (e.g., whether there is a *MECOM* rearrangement or not), it creates confusion among clinicians and pathologists and adversely impacts patient care, if the underlying systematic reason of the discrepancy due to the strengths and limitations of the different tests cannot be elucidated. In the literature, there is a knowledge gap in the systematic studies comparing OGM and targeted RNA-Seq. The aim of this study is to address this knowledge gap for comparative studies between OGM and targeted RNA-Seq. In this single institutional retrospective study, we evaluated the performance of a 108-gene anchored-multiplex-PCR-based RNA-Seq panel and compared the results with those of Optical Genome Mapping (OGM) in 467 acute leukemia cases.

## 2. Materials and Methods

### 2.1. Patients

A total of 467 acute leukemia cases were identified from our institutional database between 1 October 2023 and 31 January 2025. The cohort included 360 cases of acute myeloid leukemia (AML), 89 cases of B-acute lymphoblastic leukemia (B-ALL), 12 cases of T-ALL, and 6 cases of mixed phenotype acute leukemia (MPAL). Clinicopathologic and laboratory data were gathered through an electronic medical chart review. All cases underwent testing using both the 108-gene targeted RNA-Seq panel and OGM. For these genes, information regarding DNA-level gene rearrangements (identified by OGM) and RNA-level gene fusions (identified by NGS) was extracted. This study received approval from the Institutional Review Board and adhered to the guidelines of the Declaration of Helsinki.

### 2.2. OGM Analysis

OGM was conducted on fresh BM aspirate specimens according to the manufacturer’s instructions (Bionano Genomics, San Diego, CA, USA). The OGM procedure includes ultra-high-molecular-weight genomic DNA extraction, DNA labeling, DNA molecule imaging, genome construction, and data analysis, as has been described previously [16]. Genomic assessment was based on Genome Reference Consortium Human Build 38 (GRCh38/hg38). Data analysis was performed through Rare Variant Analysis Pipeline using the Bionano Access software (version 1.8.2) and/or VIA (version 7.1), applying two sets of Feature files, the HemeTargets and hg38-primary transcripts [16,18].

### 2.3. Targeted RNA-Seq

RNA was extracted from peripheral blood or bone marrow aspirate specimens. The anchored multiplex PCR (AMP) method was utilized as a target enrichment method combined with next-generation sequencing (NGS) to identify fusion transcripts in the 108 genes listed in the Table S1. The RNA-based assay targets exons of either one or both translocated genes in chimeric transcripts reported in hematologic malignancies. The AMP chemistry utilizes unidirectional gene-specific primers (GSP2), targeting at least one of the two gene partners involved in the translocation to allow for the capture of novel fusion partners. Amplified targets were sequenced using bidirectional sequencing on an Illumina sequencer, and the sequencing reads are aligned to the human reference genome GRCh37/hg19. Fusion transcripts were identified using Archer Analysis Software v6.2.7. [38].

### 2.4. Variant Interpretation

OGM and RNA-Seq results were classified according to the 2019 American College of Medical Genetics and Genomics (ACMG) and Clinical Genome Resource (ClinGen) Guidelines [39] and 2017 Association of Molecular Pathology (AMP), American Society of Clinical Oncology (ASCO), and College of American Pathologists (CAP) Standards and Guidelines for the Interpretation and Reporting of Sequence Variants in Cancer [40] into three tiers, respectively. Tier 1 variants are SVs or CNVs with established diagnostic, prognostic, or therapeutic relevance. These variants are acknowledged in clinical practice guidelines, e.g., National Comprehensive Cancer Network (NCCN), are defined by current World Health Organization Classification (WHO-HAEM5) or International Consensus Classification (ICC) criteria, or are the target of FDA-approved treatments. Tier 2 variants have some clinical relevance but do not satisfy all the criteria for Tier 1. Tier 3 variants are acquired aberrations without a known link to neoplastic disorders.

### 2.5. Statistical Analysis

Minitab software (version 18) was utilized for statistical analyses. A Fisher’s exact test and/or Chi-square test were employed for categorical variables. A *p*-value of less than 0.05 was considered statistically significant.

## 3. Results

### 3.1. OGM and RNA Seq Analysis Revealed Clinically Significant Gene Rearrangements and Fusions

**Figure 1:**
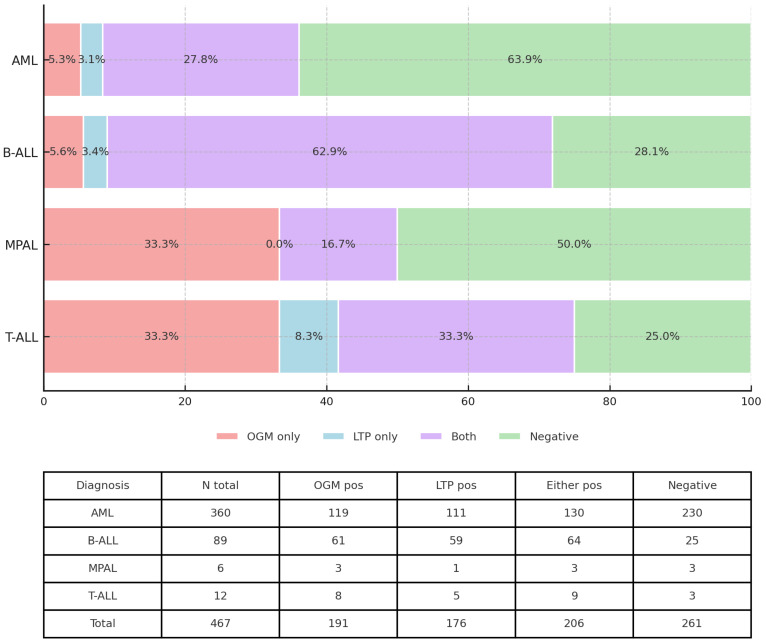
Detection of genomic aberrations across hematologic malignancies. Horizontal 100% stacked bars show, for each diagnosis (AML, B-ALL, MPAL, and T-ALL), the proportion of cases in which an aberration was identified only by optical genome mapping (OGM; red), only by targeted RNA fusion testing (LTP; blue), by both methods (purple), or by neither method (Negative; green). Percent values are displayed within segments. The table beneath the bars lists the per-diagnosis totals: number of cases and counts detected by OGM, by LTP, by both methods, and negative cases.

OGM and/or RNA seq revealed at least one gene rearrangement/fusion in 206 of 467 (43.6%) acute leukemia cases. This detection rate varied significantly across different types of acute leukemia, ranging from 36.1% in AML to 75% in T-ALL (*p* < 0.001) (Figure 1).

**Figure 2:**
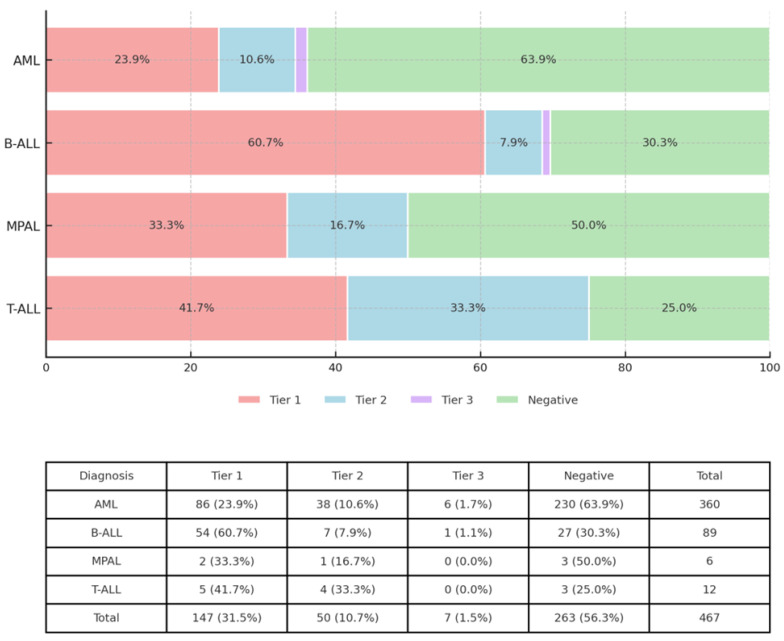
Detection of genomic aberrations according to tier across hematologic malignancies. Horizontal 100% stacked bars show, for each diagnosis (AML, B-ALL, MPAL, and T-ALL), the proportion of cases in which an aberration was identified as Tier 1 (red), Tier 2 (blue), Tier 3 (purple), or Negative (green). Percent values are displayed within segments ≥ 5% to retain numerical detail. The table beneath the bars lists per-diagnosis totals: number of cases and counts stratified by Tier.

A clinically significant Tier 1 aberration was observed in 147 of 467 (31.5%) cases. Tier 1 detection rates varied substantially across acute leukemia types, ranging from 60.7% in B-ALL to 23.9% in AML (*p* < 0.001) (Figure 2).

Rearrangement of *KMT2A* and *MECOM* were the most common Tier 1 aberrations in AML, whereas *BCR::ABL1* and *BCL11B* rearrangement were the most common aberrations in B-ALL and T-ALL, respectively. Case-level data are available in Table S2.

### 3.2. OGM and RNA-Seq Results Were Concordant in Most Cases

**Figure 3:**
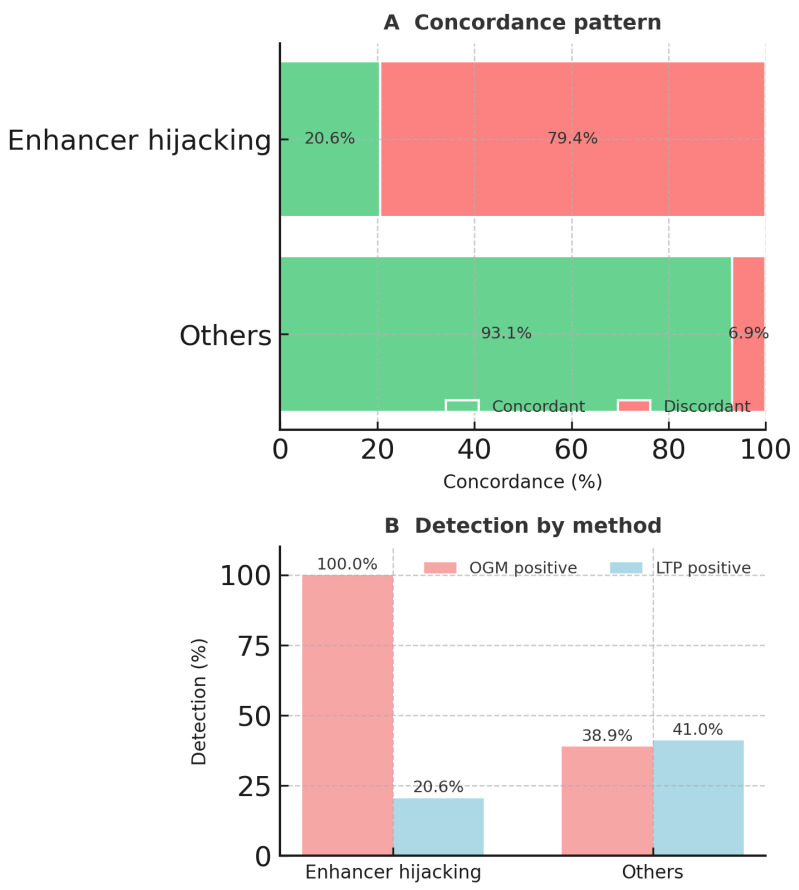
Concordance and assay detection for enhancer-hijacking vs. other aberrations. (**A**)—Concordance pattern; 100% stacked bars compare concordant calls (green) with discordant calls (red) between optical genome mapping (OGM) and targeted RNA fusion testing (LTP). Enhancer-hijacking lesions, such as *MECOM-R*, *BCL11B-R*, *CDK6::MNX1*, and *IGH-R*, show markedly lower concordance (20.6%) than all other aberrations (93.1%). (**B**)—Detection by method. Bars display the percentage of rows detected positive by OGM (red) and by LTP (blue) for each category. OGM detects 100% of enhancer-hijacking events, whereas LTP detects only 20.6%.

Of 234 gene/rearrangement fusions, 175 (74.7%) were concordantly detected by OGM and RNA-Seq. The concordance rate varied across different leukemia types, ranging from 80.2% in B-ALL to 41.7% in T-ALL (*p* < 0.001) (Table 1). Among the Tier 1 aberrations, all rearrangements/fusions, including *ABL1*, *ABL2*, *CBFB*, *NUP98*, *RARA*, *RUNX1*, *TCF3*, *JAK2*, *MEF2D*, and *MLLT10*, were concordant (Table 1). Thirty-seven of 234 (15.8%) aberrations were only detected by OGM, whereas 22 (9.4%) were detected only by RNA-Seq (Table 2). Enhancer-hijacking lesions, such as *MECOM*-R, *BCL11B-R*, *CDK6::MNX1*, and *IGH* rearrangements, showed markedly lower concordance (20.6%) than all other aberrations (93.1%) (*p* < 0.001) (Figure 3). All *BCL11B* and 73.9% of *MECOM* rearrangements were detected by OGM and not detected by RNA-Seq (Table 2).

Conversely, gene fusions arising from intrachromosomal recombination are frequently interpreted as deletions rather than as gene rearrangements or fusions by OGM. Most such events, e.g., *EPS15L1::KLF2*, *FAR2::CCND2*, *LMO1::RIC3*, *CDKN2A::SLC24A2*, *NF1::RHOT1*, and *IKZF2::LOC102725082*, are classified as Tier 3 variants. However, Tier 1 and Tier 2 rearrangements also occur through this mechanism, including deletions on chromosome 9, leading to *SET::NUP214* and *NUP214::ABL1*, and on chromosome X/Y, involving *P2RY8::CRLF2*. Interestingly, several *KMT2A* partial tandem duplications (*KMT2A*-PTD) also showed discordance: two were detected only by OGM, and others only by RNA-Seq (see case-level data in Table S2).

## 4. Discussion

Overall, targeted RNA sequencing and OGM results were concordant in most cases within this cohort. However, approximately 10% of cases showed discordance between OGM and RNA-Seq results. The primary reason for this discrepancy is that OGM detects genomic aberrations, whereas RNA-Seq remains negative due to enhancer hijacking of the involved genes. Conversely, discordant cases where RNA-Seq is positive and OGM is negative typically involve gene insertions, of which the inserted gene could not be identified by OGM, or intrachromosomal deletions, in which the impact of deletion for fusion formation was not appreciated in the context of routine analysis.

There is an important distinction between cytogenetic assays and molecular assays. Cytogenetic assays, such as chromosome analysis, FISH, and OGM, primarily assess flanking regions rather than the exact fusion junction. With cytogenetic methods, the fusion junction at the base-pair level is inferred or estimated rather than directly observed. In contrast, molecular assays, including both DNA- and RNA-based methods, focus on direct observation of the fusion junction at single-base resolution. However, except for long-read sequencing assays, molecular approaches typically provide very limited information about the flanking regions of fusions. Furthermore, RNA-level clinical assays focus on the detection of a chimeric fusion transcript rather than the overexpression of a gene. The main pathogenic mechanism in enhancer hijacking is overexpression of the “hijacked” gene rather than the formation of a fusion transcript [41].

In AML, discrepancies in detecting *MECOM* rearrangements were particularly pronounced; a substantial majority of *MECOM* rearrangements identified by OGM were undetected by RNA sequencing. *MECOM* exemplifies enhancer hijacking as a mechanism of gene dysregulation. *MECOM*, located on chromosome band 3q26, generates multiple transcript variants through distinct transcription initiation sites and intergenic alternative splicing [42]. These variants include EVI1, the longer transcript MDS1-EVI1, and a truncated, non-oncogenic form called EVI1Δ324 [43]. EVI1 acts as an oncogenic transcription factor [44,45], and its abnormal expression in malignant myeloid cells disrupts myeloid differentiation, perturbs cell-cycle regulation, and alters cellular signaling pathways [46]. Notably, chromosomal rearrangements involving *MECOM* typically lead to selective enhancer-driven overexpression of EVI1, without similarly affecting expression levels of the MDS1-EVI1 isoform. Our results showed that unless *MECOM* rearrangement also leads to a chimeric fusion transcript, such as *NRIP::MECOM* or *ETV6::MECOM*, presumably driven by the promoter of the fused genes, such as *NRIP1* or *ETV6*, RNA-Seq in routine clinical practice often fails to detect *MECOM* rearrangements.

For B-ALL, the major discrepancies involve the immunoglobulin heavy-chain locus at 14q32. The *IGH* cluster contains strong enhancers that normally are dedicated to driving antibody gene expression in B-cells. In lymphoid cancers, however, these enhancers often end up fused with other genes. The classic scenario is in mature B-cell lymphomas, such as Burkitt lymphoma, in which the *IGH* enhancer activates MYC, or follicular lymphoma, in which the *IGH* enhancer activates BCL2. In cases of B-ALL, similar translocations occur, including *IGH::CRLF2*, *IGH::EPOR*, and *IGH::CEBPA*.

Enhancer hijacking also involves *BCL11B*, which encodes a transcription factor essential for T-cell development. Under physiological conditions, *BCL11B* expression is tightly restricted to the T-cell lineage. *BCL11B* is expressed at the point of thymocyte commitment, helps to enforce T-lineage identity, and is *not* expressed in multipotent hematopoietic stem/progenitor cells (HSPCs) or in myeloid or B-cell lineages. However, *BCL11B* is often aberrantly activated due to structural variations that put *BCL11B* under the control of enhancers active in HSPCs or early precursors, which effectively results in the overexpression of *BCL11B* in MPAL or ETP-ALL [47]. In addition, *BCL11B* enhancers may be hijacked by *TLX1* and *TLX3* in more mature T-cell malignancies (T-ALL), resulting in the overexpression of *TLX1* and *TLX3* [48]. Since no chimeric fusion transcript is generated, aberrations involving *BCL11B* are not detected by RNA-Seq.

Small gene insertions and intrachromosomal deletions are among the primary causes of false-negative results in optical genome mapping (OGM). Small insertions leading to gene fusions are especially challenging for cytogenetic assays, as they often evade detection due to their limited size and subtle structural impact. In OGM, their detection typically requires prior knowledge of the fusion event, since their small size falls below the resolution threshold. A representative example is the *KMT2A::AFDN* fusion, due to insertion, which has been presented elsewhere as a case report [49].

Several intrachromosomal deletions resulting in gene fusions were not initially appreciated in the OGM analysis. A well-known example of an intrachromosomal deletion resulting in a gene fusion is the *CHIC2* deletion leading to the *FIP1L1::PDGFRA* fusion [50]. However, our data suggest that chromosome 4q12 is not the only locus where this mechanism is involved. Deletions at chromosome Xp11 resulting in *P2RY8::CRLF2* and at chromosome 9q34 resulting in *NUP214::ABL1* and *SET::NUP214* are additional examples of functionally significant intrachromosomal deletions that should be carefully assessed in OGM analysis.

*KMT2A* partial tandem duplication (*KMT2A*-PTD) represents an intriguing genomic alteration. Although the overall concordance between optical genome mapping (OGM) and RNA-Seq for *KMT2A*-PTD is high, a subset of discordant cases was identified. Notably, these discordant cases were evenly divided: half were detected exclusively by OGM, and the other half solely by RNA-Seq. RNA-Seq–positive but OGM–negative cases may reflect the limited resolution of OGM for small duplications, whereas OGM–positive but RNA-Seq–negative cases could represent non-expressed or non-functional duplications that do not produce detectable fusion transcripts.

This study has several limitations. First, it is a single-institution study conducted at a major academic center focused on adult malignancies; therefore, the findings may not be generalizable to clinical laboratories or healthcare settings with more limited resources. Second, to ensure a fair “apples-to-apples” comparison, we restricted our analysis to structural variants involving the 108 genes included in the targeted RNA fusion panel. One could argue that this approach does not fully capture the broader analytical and clinical capabilities of OGM, which can detect a wider range of structural variants and copy number variants. Third, we assessed the performance of targeted RNA-NGS relative to karyotyping and FISH, without including other relevant assays, such as other RNA-based NGS panels, whole-genome sequencing (WGS), or array-based copy number analysis.

Despite its limitations, this study is notable for its large cohort size and provides valuable insights into the limitations of RNA sequencing (RNA-seq) in detecting oncogenic rearrangements that lead to overexpression through enhancer-hijacking events that typically do not produce chimeric fusion transcripts. Conversely, the functional impact of intrachromosomal deletions that result in gene rearrangements or fusion transcripts should be carefully evaluated using OGM.

## 5. Conclusions

For rearrangements involving *MECOM* in AML, *IGH* in B-ALL, and *BCL11B* in T-ALL, alternative technologies such as FISH (particularly in resource-limited settings), optical genome mapping (OGM), or whole-genome sequencing (WGS), especially long-read sequencing, should be considered, as these aberrations frequently yield false-negative results on RNA-seq panels. The functional impact of intrachromosomal deletions that result in gene rearrangements or fusion transcripts should be carefully evaluated using OGM, particularly for genes such as *NUP214* and *CRLF2*, located at chromosomes 9q34 and Xp11, respectively.

## Figures and Tables

**Table 1:** Distribution of detected gene rearrangements/fusions according to diagnosis, tier, and concordance (based on clinical reports).

AML						B-ALL (Continued)					
Aberration	Tier	N	Concordant	Discordant	Concordance	Aberration	Tier	N	Concordant	Discordant	Concordance
*KMT2A-R*	I	22	21	1	95.5	*ABL2-R*	I	2	2	0	100
*MECOM-R*	I	22	6	16	27.3	*JAK2-R*	I	2	2	0	100
*CBFB::MYH11*	I	16	16	0	100	*MEF2D-R*	I	2	2	0	100
*NUP98-R*	I	5	5	0	100	*IGH::P2RY8*	I	1	1	0	100
*PML::RARA*	I	5	5	0	100	*PAX5alt*	I	1	1	0	100
*RUNX1::RUNX1T1*	I	5	5	0	100	*PICALM::MLLT10*	I	1	1	0	100
*DEK::NUP214*	I	4	4	0	100	*IGH::BCL2*	I	1	0	1	0
*CBFA2T3::GLIS2*	I	2	2	0	100	*P2RY8::IGH AS*	I	1	0	1	0
*BCR::ABL1*	I	1	1	0	100	*IKZF1 del*	II	23	23	0	100
*KAT6A::CREBBP*	I	1	1	0	100	*IGH::CEBPA*	II	2	1	1	50
*PICALM::MLLT10*	I	1	1	0	100	*KMT2A-PTD*	II	1	1	0	100
*BCL11B-R*	I	1	0	1	0	*IGH::CEBPB*	II	1	0	1	0
*CDK6::MNX1*	I	1	0	1	0	*SET::NUP214*	II	1	0	1	0
*KMT2A-PTD*	II	30	26	4	86.7	*IKZF2-R*	III	1	0	1	0
*Variant RUNX1*	II	5	3	2	60	*NF1::RHOT1*	III	1	0	1	0
*NPM1::MLF1*	II	1	1	0	100	Positive		86	69	17	80.2
*ETV6-R*	II	1	0	1	0	Negative		27	27	0	100
*IKZF1 del*	II	1	0	1	0	Total (B-ALL)		113	96	17	85
*SET::NUP214*	II	1	0	1	0	**MPAL**					
*CSTF3::WT1*	III	1	1	0	100	*BCR::ABL1*	I	1	1	0	100
*IKZF1::LRBA*	III	1	1	0	100	*MECOM-R*	I	1	0	1	0
*KPNA1::TP63*	III	1	1	0	100	*ETV6-R*	II	1	0	1	0
*CDKN2A::SLC24A2*	III	1	0	1	0	*Positive*		3	1	2	33.3
*EPOR-R*	III	1	0	1	0	*Negative*		3	3	0	100
*EPS15L1::KLF2*	III	1	0	1	0	Total (MPAL)		6	4	2	66.7
*FAR2::CCND2*	III	1	0	1	0	**T-ALL**					
*LMO1::RIC3*	III	1	0	1	0	*BCL11B-R*	I	5	0	5	0
Positive		133	100	33	75.2	*ETV6-R*	II	4	3	1	75
Negative		230	230	0	100	*NUP214::ABL1*	II	2	1	1	50
Total (AML)		363	330	33	90.9	*SET::NUP214*	II	1	1	0	100
						Positive		12	5	7	41.7
**B-ALL**						Negative		3	3	0	100
*BCR::ABL1*	I	22	22	0	100	Total (T-ALL)		15	8	7	53.3
*IGH::CRLF2*	I	6	2	4	33.3						
*KMT2A-R*	I	4	4	0	100	**TOTAL**					
*TCF3-R*	I	4	4	0	100	Positive		234	175	59	74.7
*ETV6::RUNX1*	I	3	3	0	100	Negative		263	263	0	100
*IGH::EPOR*	I	2	2	0	100	Grand Total		497	438	59	88.1
*P2YR8::CRLF2*	I	3	0	3	0						

**Table 2:** Distribution of detected gene aberrations/fusions according to the tier and methodology (based on clinical reports).

Aberration	Tier	OGM Only	LTP Only	Both	Total	Aberration	Tier	OGM Only	LTP Only	Both	Total
*KMT2A-R*	I	0 (0.0%)	1 (3.8%)	25 (96.2%)	26	*KMT2A-PTD*	II	2 (6.5%)	2 (6.5%)	27 (87.1%)	31
*BCR::ABL1*	I	0 (0.0%)	0 (0.0%)	24 (100.0%)	24	*Z*	II	0 (0.0%)	1 (4.3%)	22 (95.7%)	23
*MECOM-R*	I	17 (73.9%)	0 (0.0%)	6 (26.1%)	23	*ETV6-R*	II	1 (16.7%)	2 (33.3%)	3 (50.0%)	6
*CBFB::MYH11*	I	0 (0.0%)	0 (0.0%)	16 (100.0%)	16	*Variant RUNX1*	II	1 (20.0%)	1 (20.0%)	3 (60.0%)	5
*BCL11B-R*	I	6 (100.0%)	0 (0.0%)	0 (0.0%)	6	*SET::NUP214*	II	0 (0.0%)	2 (66.7%)	1 (33.3%)	3
*IGH::CRLF2*	I	4 (66.7%)	0 (0.0%)	2 (33.3%)	6	*IGH::CEBPA*	II	1 (50.0%)	0 (0.0%)	1 (50.0%)	2
*NUP98-R*	I	0 (0.0%)	0 (0.0%)	5 (100.0%)	5	*NUP214::ABL1*	II	0 (0.0%)	1 (50.0%)	1 (50.0%)	2
*PML::RARA*	I	0 (0.0%)	0 (0.0%)	5 (100.0%)	5	*IGH::CEBPB*	II	1 (100.0%)	0 (0.0%)	0 (0.0%)	1
*RUNX1::RUNX1T1*	I	0 (0.0%)	0 (0.0%)	5 (100.0%)	5	*NPM1::MLF1*	II	0 (0.0%)	0 (0.0%)	1 (100.0%)	1
*DEK::NUP214*	I	0 (0.0%)	0 (0.0%)	4 (100.0%)	4	*CDKN2A::SLC24A2*	III	0 (0.0%)	1 (100.0%)	0 (0.0%)	1
*TCF3-R*	I	0 (0.0%)	0 (0.0%)	4 (100.0%)	4	*CSTF3::WT1*	III	0 (0.0%)	0 (0.0%)	1 (100.0%)	1
*ETV6::RUNX1*	I	0 (0.0%)	0 (0.0%)	3 (100.0%)	3	*EPOR-R*	III	0 (0.0%)	1 (100.0%)	0 (0.0%)	1
*IGH::EPOR*	I	2 (66.7%)	1 (33.3%)	0 (0.0%)	3	*EPS15L1::KLF2*	III	0 (0.0%)	1 (100.0%)	0 (0.0%)	1
*P2YR8::CRLF2*	I	0 (0.0%)	3 (100.0%)	0 (0.0%)	3	*FAR2::CCND2*	III	0 (0.0%)	1 (100.0%)	0 (0.0%)	1
*ABL2-R*	I	0 (0.0%)	0 (0.0%)	2 (100.0%)	2	*IKZF1::LRBA*	III	0 (0.0%)	0 (0.0%)	1 (100.0%)	1
*CBFA2T3::GLIS2*	I	0 (0.0%)	0 (0.0%)	2 (100.0%)	2	*IKZF2 = R*	III	0 (0.0%)	1 (100.0%)	0 (0.0%)	1
*JAK2-R*	I	0 (0.0%)	0 (0.0%)	2 (100.0%)	2	*KPNA1::TP63*	III	0 (0.0%)	0 (0.0%)	1 (100.0%)	1
*MEF2D-R*	I	0 (0.0%)	0 (0.0%)	2 (100.0%)	2	*LMO1::RIC3*	III	0 (0.0%)	1 (100.0%)	0 (0.0%)	1
*PICALM::MLLT10*	I	0 (0.0%)	0 (0.0%)	2 (100.0%)	2	*NF1::RHOT1*	III	0 (0.0%)	1 (100.0%)	0 (0.0%)	1
*CDK6::MNX1*	I	1 (100.0%)	0 (0.0%)	0 (0.0%)	1						
*IGH::BCL2*	I	1 (100.0%)	0 (0.0%)	0 (0.0%)	1	Total		37 (15.8%)	22 (9.4%)	175 (74.7%)	234
*IGH::P2RY8*	I	0 (0.0%)	0 (0.0%)	1 (100.0%)	1						
*KAT6A::CREBBP*	I	0 (0.0%)	0 (0.0%)	1 (100.0%)	1						
*P2RY8::IGH AS*	I	0 (0.0%)	1 (100.0%)	0 (0.0%)	1						
*PAX5alt*	I	0 (0.0%)	0 (0.0%)	1 (100.0%)	1						

## Data Availability

Data are contained within the article.

## Supplementary Materials

The following supporting information can be downloaded at https://www.mdpi.com/article/10.3390/cancers17213458/s1: Table S1: 108 Analyzed Genes; Table S2: Case Level Data.
